# Battery Charger Prototype Design for Tire Pressure Sensor Battery Recharging

**DOI:** 10.3390/s19010124

**Published:** 2019-01-02

**Authors:** C. Bambang Dwi Kuncoro, Min-Feng Sung, Yean-Der Kuan

**Affiliations:** 1 Graduate Institute of Precision Manufacturing, National Chin-Yi University of Technology, Taichung 41170, Taiwan; kuncoro.bambang@polban.ac.id; 2 Electrical and Instrumentation Laboratory, Politeknik Negeri Bandung, Bandung 40012, Indonesia; 3 Kenda Rubber Ind. Co. Ltd., Yuanlin 51064, Taiwan; bigeyes@kenda.com.tw; 4 Department of Refrigeration, Air Conditioning and Energy Engineering, National Chin-Yi University of Technology, Taichung 41170, Taiwan

**Keywords:** wireless, charger, battery, pressure sensor, tire, automotive

## Abstract

One of the important devices in a vehicle is the tire pressure sensor. This device couples with other instruments inside the vehicle assisting the drivers in knowing the correct information about their vehicle’s tire pressure. This information helps improve vehicle handling, increases gas mileage, and extends tire lifespan. Once mounted inside the tire, the tire pressure sensor is a stand-alone device. It is powered by a battery that has a limited operating life. Due to it being mounted inside the tire, the driver does not frequently check tire pressure sensor battery. If the battery runs out, battery replacement is not an effective option. This work presents a battery charging prototype that recharges the tire pressure sensor battery. The developed device uses electromagnetic principles to wirelessly transmit power to a device that needs power. We designed a prototype and conducted some laboratory scale experiments. Experimental and validation were based on a tire pressure sensor developed by Kenda Rubber Ind. Co., Ltd, Taiwan (R.O.C). This tire pressure sensor consumes power from a 4.8 V 700 mAh Li-ion rechargeable battery. The experimental results show that the prototype can transmit 4.9 V induction voltage. The maximum current is up to 850 mA with the optimum transmission distance at around of 1.5 cm. This prototype recharges the tire pressure sensor battery wirelessly to extend its battery-power life.

## 1. Introduction

Today vehicle functionality goes well beyond basic transportation. Engineers have been finding ways of integrating technology into vehicles since they were manufactured [1]. The most important purposes of added vehicles technology are significant improvements in functionalities, automation, performance, comfort, and safety while driving. At the same time, the decreasing cost of electronic technology allows devices to be used to support numerous functions in vehicles. The sensor is one of the essential elements in any monitoring system. This is particularly true for road vehicles when introducing some form of assistance to the driver. In recent years, much progress and improvements have been made in vehicle safety automotive electronics.

The tire pressure sensor is an example of how electronic systems are making vehicles safer. The tire pressure sensor consists of a sensor that sends signals to a receiver connected to a small electronic processor that provides tire pressure information to the vehicle driver [2]. The vehicle driver receives information on the correct vehicle tires pressure to improves handling, extends lifespan, and increase gas mileage.

Until now, many tire pressure sensor systems have been developed with different architecture configurations from various research institutions, research universities and companies. One of the most widely used systems was implemented as a factory-installed feature on high-end vehicles. A tire Pressure Monitoring System (TPMS) was first developed in 1997 [3]. It is just one of many rapidly-growing applications in automotive technology. TPMS is a device that can be used to continuously monitor vehicle tires air pressure and provide the driver with tire pressure information and an alert signal if an anomaly occurs in one or more tires. The driver can use that alert signal as a reference for their vehicle tires condition. This plays an important role in improving safety during driving, while concurrently improving traction control, decrease fuel consumption, reduce tire wear, and increase braking efficiency [4]. If the driver drives a vehicle with flat tires, TPMS is the proper device to monitor incorrect tire air pressure conditions.

Once mounted in the tire, the tire pressure sensor is a stand-alone device. It is powered by a battery that has a limited operating life. Most tire pressure sensors use Lithium (Li)-ion batteries as the power source. A small Li-ion battery shows a distinguished energy capacity that refers to the weight ratio with an output voltage of about 3.7–4.8 V (open-circuit), as well as a higher operating temperature range [5]. In the tire pressure sensor application case, if the tire pressure sensor module battery runs out, battery replacement is not only an ineffective option, but it will also raise some very serious environmental issues regarding the end-of-life battery recycling.

Several technical approaches can be used to extend the operation of any battery operated in the tire pressure sensor module. Some research activities were already done regarding the battery operating life for tire pressure sensor. T. Lange et al. considered using a low-power component in the module design process [5]. They argued that it is very important to consider and select low-power components. This can be achieved if the components have very low current consumption in sleep or standby mode. The operating system can also be employed to control variable clock frequency and operating voltage to increase battery life in modern ICs [6]. The lifetime of any battery-operated device can be extended by battery management. However, this method needs an additional circuit to offset reducing operating voltage. Moreover, this method also influences the device performance. Currently, the solution for tire pressure monitoring problems in tire modules can be improved using microcontrollers because it can be performed in a system that has a significant role in power management [7]. Another effective way to extend battery life is to use energy harvesting techniques because of small power demand, efficiency, and inexpensive power sources [8]. This technique is a process that captures and stores an amount of energy from ambient or external sources such as solar power, thermal energy, wind energy, water energy, and kinetic energy. These methods have been used in macroscale devices. With the newest developments in the low power requirements of battery-operated devices such as the tire pressure sensor module, and this technique has become one potential solution in practical applications. One of the other alternative techniques, such as inductive coupling power method, will be developed in this work. This technique needs to be studied and investigated to meet practical applications regarding prolonging tire pressure sensor battery operation life. This research work is our preliminary study applied to a vehicle technology Wireless Power Transmission (WPT) system. We use this method (WPT) to transmit electrical energy without cable interconnection from a power source to the load (battery) over a distance. The objective of this work is to develop a prototype wireless power charger that has main functions is to recharge a tire pressure sensor battery whenever the battery needs power. We designed a prototype and performed some laboratory scale experiments to validate the prototype performance using a tire pressure sensor developed by Kenda Rubber Ind. Co., Ltd, Taiwan (R.O.C). The proposed technique is quite different from other published methods. Adopting the magnetic induction principle, an amount of energy can be transmitted over an air gap without a core from the power source to the load. In the next research work regarding tire pressure sensor power recharging application, we plan to implement the prototype in a real operating environment where the battery target location is inside the vehicle tire. The battery is connected to a wireless receiver module where the receiver coil is attached around the vehicle wheel (rotatable location). The receiver coil will then be coupled inductively to the transmitter module through a transmitter coil attached at a fixed location around the vehicle disc brake. The transmitter module connects with the vehicle accumulator as the power sources. As long as the transmitter and receiver coils are inductively coupled, the transmitter module can wirelessly recharge the tire pressure battery whenever the vehicle is either moving or stopping. Moreover, the receiver module rotational motion gives a positive effect in increasing the induction voltage at the receiver coil [9]. This domino effect is a benefit of this work because the transmitter module can generate more electromotive force induction to the receiver module.

## 2. Materials and Methods

### 2.1. Wireless Power Transmission (WPT)

In recent years, wireless power transmission systems have become fascinating as a re-emerging technology. Wireless power transmission system was developed by taking the mutual induction and resonance principle, which were developed over the past years. WPT is an electric energy propagation method that enables an electrical load to receive electromagnetic energy from a power source over a distance without the use of interconnecting wires [10,11]. The experiments of Heinrich Hertz are the first attempt to emit high-frequency wireless power transmission using parabolic reflectors in 1888 [12]. Hertz used induction coils connected by an oscillator to emit electricity over a tiny air gap. His experiment result confirmed the existence of electromagnetic radiation experimentally for the first time. The WPT system is composed of electromagnetic devices, control sub-systems, and power electronic circuits. WPT is useful in cases in which connecting lines are inconvenient, hazardous or impossible. By increasing the efficiency of the overall system, this technology is appealing to various applications because of its convenience and better user experience.

The most common wireless power transfer technology is electromagnetic induction. The fundamental WPT theorem is based on the equations of Maxwell. Two equations are known as the Ampere circuit and the Faraday induction laws [13]. The Ampere circuit law describes a correlation between electric currents and the magnetic fields generated by them. According to the Kelvin-Strokes theorem, the Ampere circuit law can also be written in the form of an integral or differential equation. A line integral of the magnetic field around of any kind closed curve *c* is a description of the Ampere circuit law in integral equation form. Suppose the electric current *i* drifts through an area *s,* which is, in turn, bounded and enclosed by curve *c*, the line integral of magnetic field *B* around curve *c* is then equal to the total current *i* drifting through area *s* enclosed by *c* can be written as [14]:

(1) $$\overset{}{\underset{c}{\oint }}B\;dl={µ}_{0}\iint_{s}^{}J\;ds={µ}_{0}\sum i$$

where µ_0_ is the magnetic constant, d*l* is an infinitesimal element of the *c* curve, and *J* is the density of free current.

Let us say that the electric current *i* drifts through a wire coming out of an area *s*, a magnetic field will be generated, creating a concentric circle of magnetic field lines in the area perpendicular to the current line. The direction of the magnetic field line follows the right-hand rule, where the magnetic field line around the current *i* is in the same direction as the curling right-hand finger, while the current *i* in the direction of the right-hand thumb. If the shape of the closed curve *c* is a circle with the radius *r* centered on the current line (wire) location, the magnetic flux density can be obtained using the line integral [14]:

(2) $$\overset{}{\underset{c}{\oint }}B\;dl=2\pi rB_{0}={µ}_{0}i$$

(3) $$B=\frac{{µ}_{0}i}{2\pi r}$$

The electromagnetic induction phenomenon is well explained by the basic law of electromagnetism (induction law of Faraday). In electromagnetic induction, if a time-varying magnetic flux is linked to a closed loop circuit it will then induce an electromotive force (emf) around the circuit. The Faraday induction law defines that the magnetic flux Φ through an area *s* is the surface integral of the magnetic field *B* passing through that area. The magnetic flux can be described as [15]:

(4) $$\Phi =\iint_{s}^{}B.ds$$

The magnetic flux rate of change is proportional to the emf induction *ε,* which is defined as [15]:

(5) $$\varepsilon =-\frac{d\Phi }{dt}$$

The law of Lenz states that a negative sign in the Equation (5) indicates the flow of induced current caused by emf has a direction opposite to the magnetic field that produces it. 

**Figure 1:**
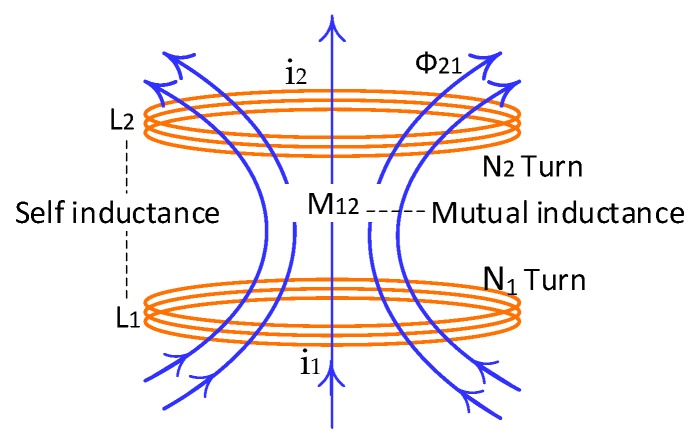
Two coils aligned in a horizontal position.

Let us see, in Figure 1, that the two coils are placed close together.

Magnetic coupling between two independent coils means the coils affect each other through the magnetic field generated by either of them. In other words, the magnetic field generated by one coil is coupled with other coils. The first coil (coil_1_) has *N*_1_ turns that carries a current *i*_1_ and emits a magnetic field vector *B*_1_. Some of the magnetic field lines through coil_1_ will also pass through coil_2_. The magnetic flux through coil_2_ due to *I*_1_ is expressed by Φ_21_. Thus, there will be an induced emf associated with the changing magnetic flux in the second coil by varying *I*_1_ with time [16]:

(6) $$\varepsilon_{21}=-N_{2}\frac{d\Phi_{21}}{dt}=-\frac{d}{dt}\iint_{coil\;2}^{}{\vec{B}}_{1}.d{\vec{A}}_{2}$$

The time rate of the current change in coil_1_ is equal to the time rate of magnetic flux change Φ_21_ in coil_2_ [17,18]:

(7) $$N_{2}\frac{d\Phi_{21}}{dt}=M_{21}\frac{di_{1}}{dt}$$

In this scenario, in addition to the self-inductance (*L*) of each coil, there exists another inductance known as mutual inductance *M*_21_. It can also be written as

(8) $$M_{21}=\frac{N_{2}\Phi_{21}}{i_{1}}$$

The mutual inductance *M*_21_ depends only on the geometrical properties of the two coils such as the number of turns, their relative spacing, and the radius of the two coils. Another mutual inductance *M*_12_ can be written as

(9) $$M_{12}=\frac{N_{1}\Phi_{12}}{i_{2}}$$

The laws of Ampere and Biot-Savart can be combined using the reciprocity theorem so that the constants are equal:

(10) $$M_{12}=M_{21}\approx M$$

While the coupling factor *k* between the self-inductance of two coils (*L*_1_ & *L*_2_) and mutual-inductance *M*_12_ can be defined as:

(11) $$k=\frac{M_{12}}{\sqrt{L_{1}L_{2}}}$$

### 2.2. Architecture Design

**Figure 2:**
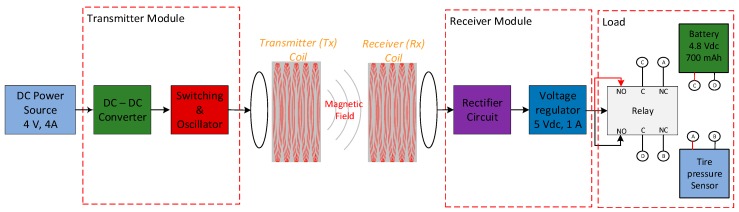
Block diagram of wireless power charging prototype for tire pressure sensor.

The proposed architecture design of the system is shown in Figure 2. It is based on inductive electromagnetic coupling. In the proposed system, the transmitter module is the primary circuit including a DC-DC converter module, the transmitter circuit and the transmitter coil, while the receiver includes the receiver coil and secondary circuit (rectifier and voltage regulator circuits). The transmitter module transmits energy to the tire pressure sensor module through the receiver coil and receiver module. The receiving voltage signal is converted to a stable output voltage of 5 V and a current maximum of 850 Amp by rectifier and voltage regulator circuits. As described in Figure 2, the output receiver module is coupled with the tire pressure sensor module through the relay switch. The relay switch changes over the connection between the battery to charger system and the battery to pressure sensor. When the charger system does not work (it does not transfer power), the normally closed contact relay connects the battery to the pressure sensor. In this state, the pressure sensor work (sensing the tire air pressure) and is powered by the battery. While, if the charger system works, the energy from the DC power source is transmitted wirelessly to the receiver module and then the relay is energized to change over relay switch from the sensor charging mode (battery and pressure sensor connected) to the battery charging mode (the battery and charger system connected). At the same time, the charging system recharges the battery power, and the air pressure sensor stops working. In a real operating environment, this prototype can work together with the TPMS module and a control system. Whenever the TPMS detects the tire pressure sensor battery critical condition, a control system will turn ON the wireless power charging of tire pressure sensor to transmit power from the transmitter module to the load (the battery of tire pressure sensor) through the receiver module.

In this research work, the primary circuit, the secondary circuit, and their coils were designed and assembled to meet the load requirements regarding the desired battery current and voltage, and the transmission distance range. The load target is 4.8 V 700 mAh Li-ion rechargeable battery. Actually, similar battery specifications can be considered, but were not used in this research work. In other words, the proposed system for wireless recharging purposes can be used for any battery-powered system that needs a maximum of voltage charger at 5 V.

### 2.3. Tire Pressure Sensor Module

**Figure 3:**
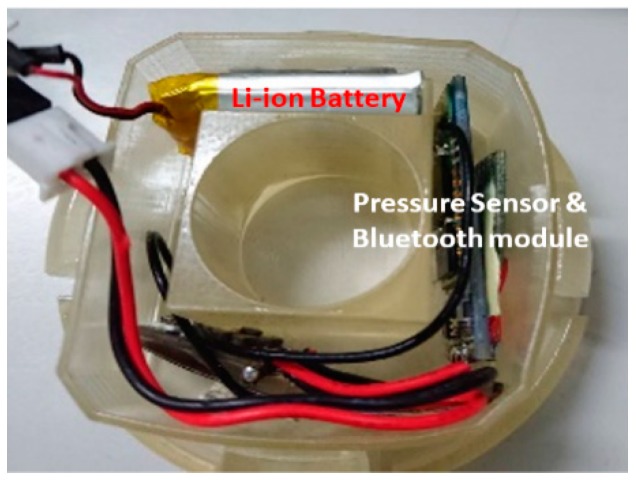
Tire pressure sensor module.

This module consists of a tire pressure sensor, Bluetooth communication module, and a battery as shown in Figure 3. The module size is 5 cm × 5 cm × 2 cm. When it is mounted inside a vehicle tire, the sensor module periodically measures internal tire pressure.

This module is powered by 4.8 V 700 mAh Li-ion rechargeable battery. The heart of this module is the air pressure sensor itself. It works based on the microcontroller. The sensor module was developed by Kenda Rubber Ind. Co., Ltd, Changhua, Taiwan (R.O.C).

### 2.4. Design and Implementation

#### 2.4.1. Coil Design

In particular, the magnetic fields are critical in wireless power charging system coils design. The coil itself, self-inductance of the coil, and the mutual inductance of two coils give a significant contribution to produce the magnetic fields. Coil design must be considered at the first wireless power charging system design step to maximize the coupling coefficient between the two coils. Some of the aspects considered include; the coil geometry, the wire type and size, the number of wire windings, and the magnetic field produced by the two coils. 

The electromagnetic properties of the primary and secondary coils must be strictly considered given the performance requirements of the proposed wireless power system. A spiral multilayer air core coil design is preferred to maximize the current (*i*) × turns (*N*) and the related magnetic field generated by the transmitter coil [8]. The self-inductance of spiral multilayer air core coil can be described by Wheeler’s approximations formula [19,20]:

(12) $$L=\frac{31.6\times N^{2}\times r_{1}{}^{2}}{\left(6\times r_{1}\right)+\left(9\times l\right)+10\times \left(r_{2}-r_{1}\right)}$$

where *L* is inductance in microHenries (µH), *N* is a total number of turns, *r*_1_ is a radius of the inner of a coil in meters, *r*_2_ is a radius of the outer coil in meters, and *l* is the length of the coil in meters.

An AWG 22 wire was chosen because of its small diameter (0.64 mm diameter and 0.0646 mm insulating layer) and can be used for carrying maximum current up to 7 Amperes. According to the calculations using Equation (12), in the case of a multilayer spiral coil having an inductance of 42.96 µH, and a wire diameter of 0.64 mm, the maximum number of wire windings and layers are 23 and 1.56, respectively. However, due to manual assembling, the transmitter coil is characterized by 2 layers and 20 turns (10 per layer), an outer diameter of 61.36 mm, an inner diameter of 60.0 mm, and 10 mm height. While the receiver coil was designed with the same diameter as the transmitter coil to maximize coupling efficiency. An AWG 28 wire was chosen in order to carry maximum current up to 2 Amperes of output voltage. In the case of a multilayer spiral coil having an inductance of 83.29 µH, and wire diameter of 0.32 mm, the maximum number of wire windings and layers are 29 and 1.67, respectively. In this implementation, the receiver coil is characterized by 2 layers and 24 turns (12 per layer), an outer diameter of 60.64 mm, an inner diameter of 60.0 mm, and 10 mm height because of manual manufacturing.

#### 2.4.2. Transmitter and Receiver Modules Design

**Figure 4:**
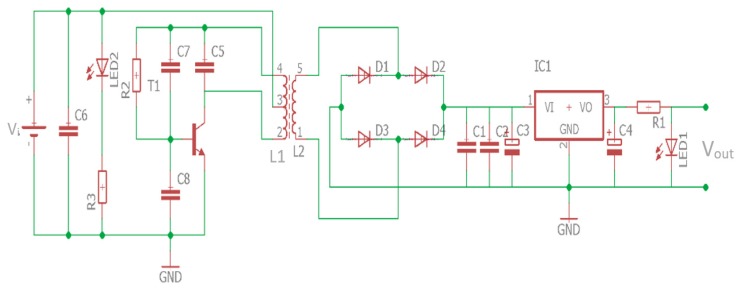
Transmitter and receiver circuits.

Once the fit coil design had been defined, a receiver and transmitter circuits were designed and implemented to fulfill the tire pressure sensor battery power requirements. The transmitter module generates power as a magnetic flux that induces the coil in the receiver module to enable the receiver circuit to collect the incoming power. The transmitter and receiver circuit for the wireless power transfer is shown in Figure 4.

**Figure 5:**
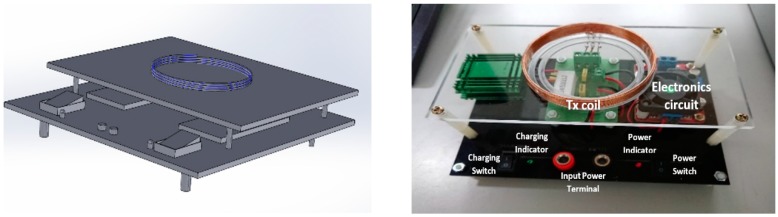
Transmitter module.

The oscillator circuit drives transmitter coil to transfer power from transmitter to receiver. The operating frequency can be described as:

(13) $$f=\frac{1}{2\pi \sqrt{LC}}$$

where *C* is the capacitance in Farad, *L* is the coil inductance in Henry, and *f* is the oscillation frequency in Hertz. In this prototype design process, we have yet to follow any WPT application standard. The prototype was designed using the operating frequency not more than 1 MHz. This operating frequency was chosen because the transmitter module of the prototype applies a switching transistor that works at the maximum operating frequency of 1 MHz. Using transmitter coil inductance is defined above (42.96 µH) and the capacitor availability in the market, a 1 nF parallel capacitor was selected to achieve operating frequency at 739 kHz. The operating frequency was calculated according to Equation (13). In the next future research work, we will design our prototype based on the Wireless Power Consortium (WPC) standard. With regard to the WPC standard, in the middle range transmission (magnetic resonance method) WPT applications, the operating frequency has to be set at 6.7 MHz. The transmitter module implementation is shown in Figure 5. The module is equipped with indicator lamps, power switch, charging switch, and power source terminals to support the module functionality.

**Figure 6:**
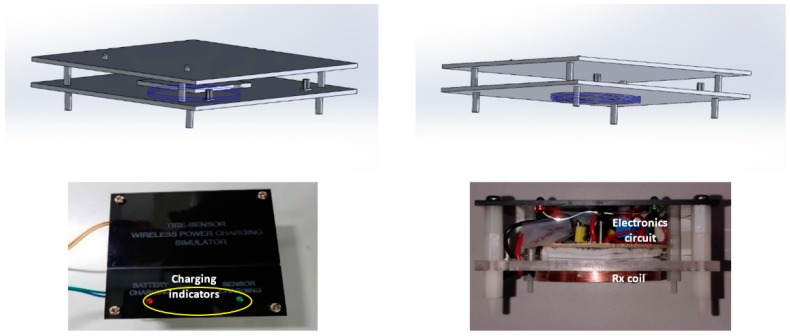
Receiver module.

The transmitter module works with 4.4 V input voltage at maximum 3.3 A. While the implemented receiver module is shown in Figure 6. The receiver module included with a rectifier circuit that has regulated output voltage of 5.0 V at a maximum current up to 850 mA.

### 2.5. Integration

**Figure 7:**
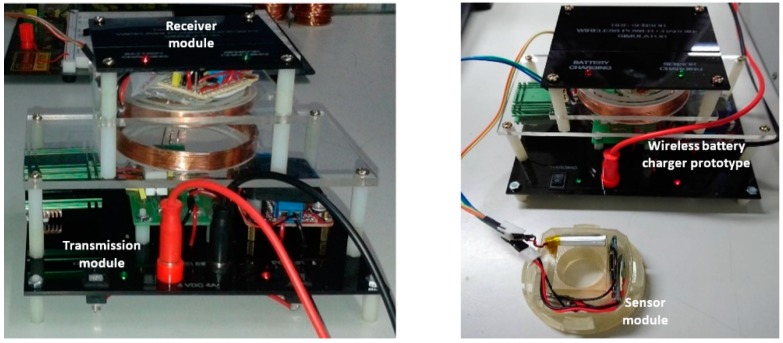
Integrated transmitter & receiver modules (**left**) and integrated wireless power charger and tire pressure sensor modules (**right**).

System integration was addressed to integrating the developed wireless recharging system with the sensor module, according to the scheme presented in Figure 1. Integration of the developed wireless recharging system is necessary to demonstrate the concept and the potentiality of the proposed system in order to recharge the battery of tire pressure sensor module. To this purpose, the coils (transmitter and receiver coils) are located to separate in such a way that their planes are in parallel to each other at horizontal alignment. The centers of both coils are located on the same axis, as shown in Figure 7 (left).

The final prototype including the wireless recharging system and tire pressure sensor module were then integrated, as shown in Figure 7 (right). The wireless recharging system integration into an existing tire pressure sensor module was challenging considering all of the electronics parts that needed to be included in the small prototype frame of the tire sensor wireless power charging simulator, as shown in Figure 7 (left). 

## 3. Results and Discussions

### 3.1. Experimental

An experiment was done to test the capability of the designed device to charge tire pressure sensor module battery wirelessly. In the experiment, DC power supply was used as a power input source. The transmitter module is part of the wireless power charger system which carries out the power transmission to the receiver module wirelessly. The receiver module will receive induction voltage and regulate the induction voltage. Furthermore, the regulated voltage will charge the tire pressure sensor module battery. 

**Figure 8:**
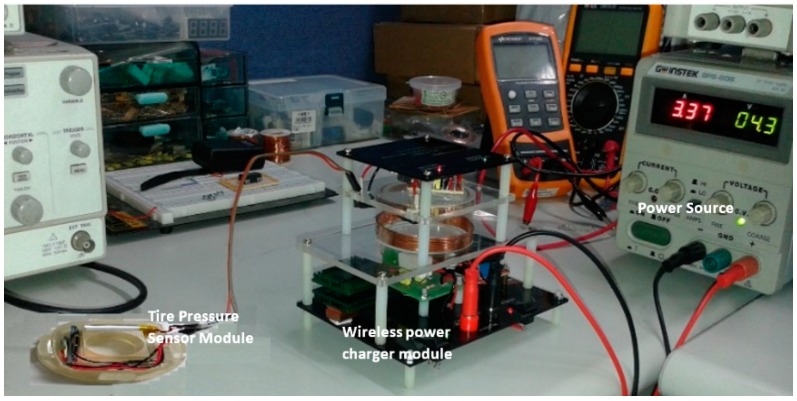
Experimental setup.

The receiver module place above the transmitter module horizontal alignment is shown in Figure 8. The power supply adjusted to around 3.3 DC Amp at 4.4 V DC to supply the power for the transmitter module. The tire pressure sensor module is connected to the output terminal of the receiver module, and it uses 4.8 V 700 mAh Li-ion rechargeable battery.

During the experiment, the designed device functions and performance were observed. The receiver module output voltage was measured, and the charging battery process was observed in 0.5 min time steps for 30 min of measurement duration.

### 3.2. Results

**Figure 9:**
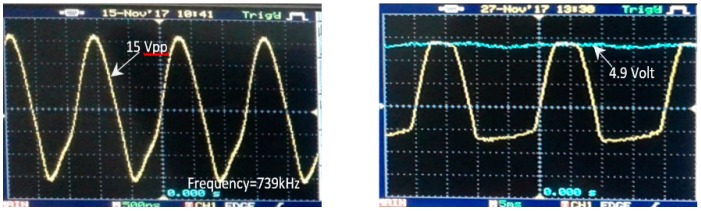
The driving voltage signal on the transmitter circuit (**left**) and the output voltage on the receiver module (**right**).

The experiments were performed to evaluate the recharging process results. The process was performed by testing and measuring the power input, the transmitter and receiver circuits signals. The transmitter module input voltage was generated by an input voltage of 4.4 V DC from a power supply. The input voltage is manipulated by the transmitter module, and then transferred wirelessly to the receiver circuit. The driving voltage on the transmitter coil is shown in Figure 9 (left).

With the current design of the wireless recharging system, a voltage of V = 15 Vpp on the transmitter coil was available with a maximum current of 3.3 A. The oscillator circuit generates the signal with operation frequency at 739 kHz. These values resulted in the final output voltage of 4.9 V (Figure 9 (right)) on the output of receiver module (open load).

**Figure 10:**
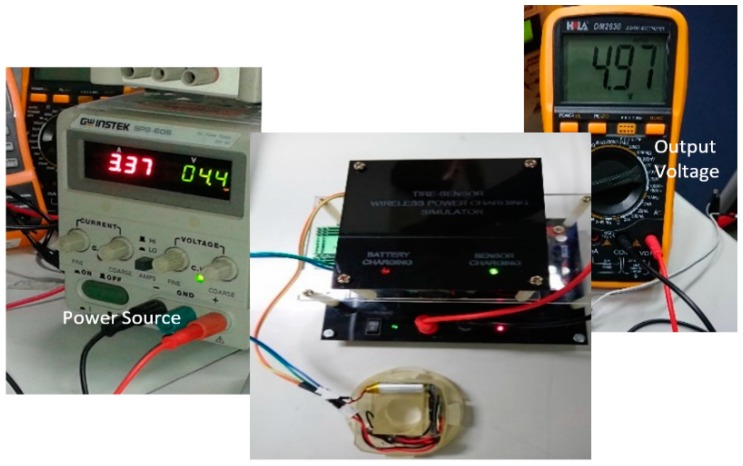
Output voltage measurement.

The regulated receiver module output voltage was measured using a multimeter, as shown in Figure 10. The optimum transmission distance between the transmitter and receiver coils is around of 1.5 cm in order to produce the output voltage of around 4.97 V DC. 

**Figure 11:**
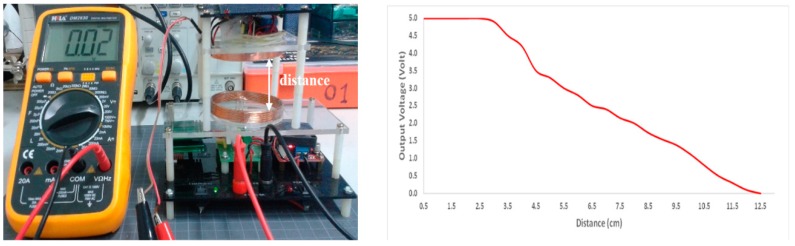
The characteristic of developed WPT measurement (**left**) and the characteristic of the open load output voltage (**right**).

The measurement of a relation between load voltage and load current with the transmission distance was performed to describe the developed WPT characteristic as shown in Figure 11 (left). Figure 11 (right) shows the characteristic of open load output voltage of developed WPT. The receiver module output voltage decreases close to inversely exponential with the increase in distance between the transmitter and receiver coils.

**Figure 12:**
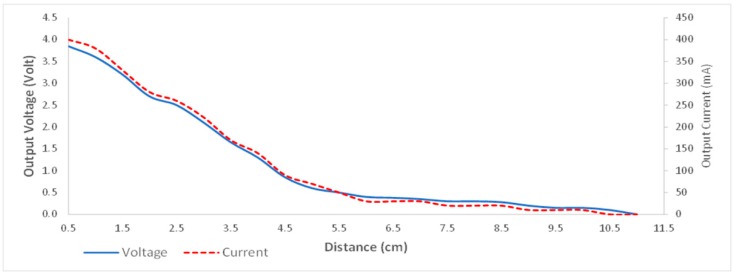
The characteristic of the output voltage and current with load of 10 Ω.

The developed WPT characteristic is also shown when the receiver module output is a closed circuit with 10 Ω resistor as shown in Figure 12. This demonstrates that increasing the distance between the WPT transmitter and the receiver coils cause the receiver module output voltage and current decrease close to inversely exponential too.

**Figure 13:**
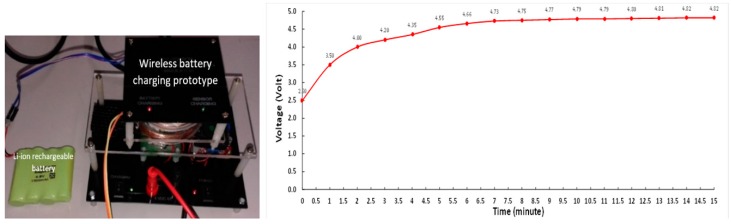
Battery charging experiment (**left**) and typical charging curve using the developed device (**right**).

The integrated hardware (Figure 13 (left)) allows the receiver module to couple with the transmitter module making wireless battery recharging. The charging curve was experimentally obtained using the wireless recharging system and measuring the battery voltage with a voltmeter, as shown in Figure 13 (right). The 4.8 V 1000 mAh Li-ion rechargeable battery was used to perform the charging curve experimental.

**Figure 14:**
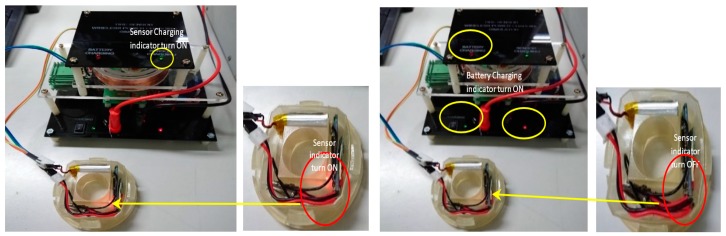
Sensor charging condition (**left**) and battery charging condition (**right**).

The charging process was also performed to determine the battery recharging functionality and the tire pressure sensor power charging process. The sensor and battery charging conditions are shown in Figure 14. As shown in Figure 14 (left), the indicator lamp on the pressure sensor and Bluetooth module light ON, and the sensor charging indicator on the receiver module also lights ON. These conditions indicate that the tire pressure sensor was charging.

While in the battery charging process as shown in Figure 14 (right), the indicator lamp on the pressure sensor and Bluetooth modules light OFF, and the indicator lamp of battery charging on the receiver and transmitter modules light ON, these conditions indicate that the battery was charging.

## 4. Conclusions

The wireless power charging will help the driver charge the tire pressure sensor battery without removing it from inside the vehicle tire. The system was designed successfully and also tested to charge the tire pressure sensor system battery developed by Kenda Co Taiwan. The experimental results show that the prototype can transmit 4.9 V induction voltage, and maximum current is up to 850 mA with the optimum transmission distance around of 1.5 cm. While this prototype has yet to be implemented in a real application (real operating environment), the experimental result is proof that the proposed concept can be accepted for the target application of this research work. This prototype can work according to the design to recharge the tire pressure sensor battery wirelessly in order to extend its battery-power life.

Since all the components utilized to develop the prototype are Commercial off-the-shelf (COTS) components that can be purchased easily from the market at low costs, there is great potential and feasibility for the prototype to be a proper battery recharger alternative method, to be scaled-up, and added in the vehicle technology in the near future.
